# Nationwide Rate of Adult ADHD Diagnosis and Pharmacotherapy from 2015 to 2018

**DOI:** 10.3390/ijerph182111322

**Published:** 2021-10-28

**Authors:** Sang-Min Lee, Hyeon-Kyoung Cheong, In-Hwan Oh, Minha Hong

**Affiliations:** 1 Department of Psychiatry, Kyung Hee University School of Medicine, Seoul 20447, Korea; maumdoctor@gmail.com; 2 Department of Internal Medicine, School of Medicine, Korea University, Ansan 15355, Korea; chongmy99@hanmail.net; 3 Department of Preventive Medicine, Kyung Hee University School of Medicine, Seoul 20447, Korea; parenchyme@gmail.com; 4 Department of Psychiatry, Myongji Hospital, Hanyang University College of Medicine, Goyang 10475, Korea

**Keywords:** adult, attention deficit hyperactivity disorder, diagnosis, pharmacotherapy

## Abstract

There is a paucity of published literature on the epidemiology of adult attention-deficit/hyperactivity disorder (ADHD). We investigated the time trends of the diagnostic and pharmacotherapy incidence of ADHD, including the first used medication, in the adult population based on a Korean population-based database from 2015 to 2018. The number of diagnosed cases of ADHD significantly increased from 7782 in 2015 to 17,264 in 2018 (*p* = 0.03), which is 0.02% to 0.04% of the total population. Similarly, the number of pharmacotherapy cases of ADHD significantly increased from 3886 in 2015 to 12,502 in 2018 (*p* = 0.01), which is 0.01% to 0.03% of total population. The most commonly used medication at the initiation of pharmacotherapy shifted from Penid in 2015 to Concerta in 2018. Furthermore, combination therapy with two or more drugs was the preferred method in 2016–2018. In conclusion, the identified diagnoses and pharmacotherapy incidences were very low, highlighting the need to improve the public’s awareness of adult ADHD.

## 1. Introduction

Attention-deficit/hyperactivity disorder (ADHD) is a common psychiatric disorder with a chronic course that can lead to negative outcomes without optimal treatment [1,2]. It was previously believed to occur only among children and adolescents. However, the concept of adult ADHD was established due to the reorganization of the Diagnostic and Statistical Manual of Mental Disorders [3], and subsequent research has been actively conducted.

The global prevalence of ADHD in children and adolescents is approximately 2–7% [4,5], and about half of cases are known to persist into adulthood [6]. In the USA, the administrative prevalence based on diagnosis ranged from 0.93 to 11.0% [7,8], while it ranged from 0.6 to 10% [9] based on prescription [10]. In the UK, the prevalence based on prescriptions ranged from 0.03 to 0.92% [11,12]. In studies outside the USA and the UK, such as in Denmark [13], Norway [14], and Australia [15], the administrative prevalence estimates were lower compared to those in the USA. Similarly, studies in Taiwan [16] and Korea [17] reported a lower prevalence. Although it is challenging to ascertain the incidence rate of mental disorders, Hong et al. investigated the diagnosis of ADHD and the transition to pharmacotherapy in children and adolescent populations by taking advantage of the mandatory universal national health insurance system in Korea [17]. They found that the diagnostic incidence was 0.3% and the pharmacotherapy incidence was 0.2%.

However, the epidemiological data of adult ADHD diagnosis and medication are limited compared to those of children and adolescents. In this study, we aimed to investigate the diagnostic and pharmacotherapeutic incidence of ADHD and conducted a trend analysis in the entire adult population (18 and above) using the National Health Insurance Services (NHIS) database.

## 2. Materials and Methods

### 2.1. Data Source and Study Population

This retrospective analysis used data from the NHIS claims database from 1 January 2014 to 31 December 2018. The inclusion criteria were as follows: (1) aged 18 and above and (2) the presence of an inpatient or outpatient medical claim containing a code for the diagnosis of ADHD (International Classification of Disease, 10the Revision code F90.0x) between 1 January 2014 and 31 December 2018, with no medication use during the 1 year preceding the claim. This study was approved by the institutional review board of Myongji Hospital (MJH 2019-05-014).

### 2.2. The Incidence of Adult ADHD Based on Diagnosis and Medication

Subjects diagnosed with ADHD (F90.0x) during a given year, but not the previous year, were defined as incident cases. The annual incidence was calculated from 2015 to 2018 by dividing the number of newly diagnosed cases of ADHD during each year by the number of person-years at risk in the NHIS dataset for the same year. The incidence of ADHD was calculated using the same method. The data on the total population aged 18 and above were obtained from the National Statistical Office (https://kosis.kr/statisticsList/statisticsListIndex.do?menuId=M_01_01&vwcd=MT_ZTITLE&parmTabId=M_01_01&outLink=Y&entrType=#content-group, accessed on 10 May 2021).

### 2.3. Other Measures

The demographic factors, such as age, sex, type of insurance, clinician specialty, and hospital level, were obtained from the NHIS database. Age was divided into the following six age groups: 18–23, 24–30, 31–40, 41–50, 51–60, and 61 years and over. The types of insurance were classified as national health insurance or medical aid. The clinicians’ specialties were categorized as psychiatry or other. The hospital levels were stratified into hospital and private clinics, and the initial ADHD medication was identified based on the list of medications (Table 1).

### 2.4. Statistical Analysis

Descriptive statistics (means and frequencies) were used to characterize the medication use and the clinical and demographic variables. To assess the trends in adult ADHD diagnosis and medication use, we examined temporal changes from 2015 to 2018 using a time series linear model. SAS 9.3 (SAS Institute Inc., Cary, NC, USA) was used to link and analyze the data. The significance level was set at *p* < 0.05.

## 3. Results

### 3.1. Diagnostic Incidence of Adult ADHD from 2015 to 2018

**Figure 1:**
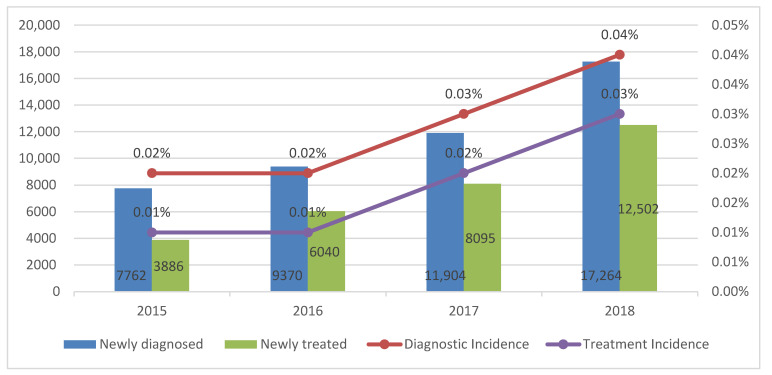
The trends of number of cases and incidence among adults with attention deficit hyperactivity disorder between 2015 and 2018.

The numbers and trends of annual incident cases are shown in Table 2 and Figure 1. The number of annual incident cases significantly increased from 7762 to 17,264 from 2015 to 2018 (*p* = 0.036). The annual diagnostic incidence during the study period also increased from 0.02% in 2015 to 0.04% in 2018. The diagnostic incidence showed an overall male predominance, though this trend gradually decreased. All the age groups, except the 31–40 year and 61 years and over groups, showed significant linear trends. New diagnoses by psychiatrists showed a statistically significant increase during the four-year period (*p* = 0.0466). 

### 3.2. Medication Rate among Newly Diagnosed Adult ADHD from 2015 to 2018

The number and trends of the annual pharmacotherapy cases are shown in Table 3 and Figure 1. The number of newly diagnosed adult patients with ADHD who initiated medication significantly increased from 3886 to 12,502 from 2015 to 2018 (*p* = 0.0196). The annual treatment incidence during the study period also increased from 0.01% in 2015 to 0.03% in 2018. The treatment incidence showed an overall male predominance, though this trend gradually decreased. All the age groups, except the 61 years and over group, showed significant linear trends.

### 3.3. First Medication Used for Adult ADHD

Most patients who initiated pharmacotherapy used two or more drugs, and the trends increased significantly (*p* = 0.02). The commonly used drugs were Penid, followed by Concerta, atomoxetine, and bupropion.

## 4. Discussion

This was the first nationwide study that investigated the diagnostic and pharmacotherapy incidence among adult patients with ADHD in Korea using nationally representative data from the NHIS.

The diagnostic and pharmacotherapy incidence in the adult population is only one-tenth that of the child and adolescent population in Korea [17]. It is worth noting that the number of diagnosed and medicated ADHD cases in the present study was limited. Thus, it does not support the debate that ADHD may be overdiagnosed and overtreated. It is noteworthy that the transition rate from diagnosis to treatment increased by 50% in 2015 and 2016, 67% in 2017, and 75% in 2018. This finding is consistent with that of the child and adolescent population [17]. The rate of pharmacotherapy in ADHD patients is known to have a wide variation ranging from 12 to 72% due to the differences in regional prescribing practice or different time frames for outcome assessment [18,19,20,21]. In Korea, however, the transition rate from diagnosis to treatment is high in all age groups. That is, once an individual is involved in clinical practice, the transition to treatment goes smoothly.

During the study period, the trends of both diagnosis and pharmacotherapy significantly increased. Although it is not possible to compare directly because of the different methods and periods of various studies, the current study found a significant increase in the trends of both diagnosis and pharmacotherapy among the adult population, consistent with studies in Taiwan [22] and Denmark [23]. However, the extent or absolute figure was remarkably low compared to that in other studies [22,23]. This is most likely due to the cultural differences that affect people’s attitudes towards psychiatric diagnosis and treatment [22], and in part by the fact that the Food and Drug Administration only approved ADHD medication for use in the adult population in September 2016.

In the trend analysis, the 18–23 years group showed a significant decrease in both diagnosis and pharmacotherapy, but the latter significantly increased in the 24–50 years group. We speculate that the 18–23 years age group are given autonomy after high school graduation and will exist outside the treatment boundary because it is a period with few compulsory tasks, such as work or school. Meanwhile, the 24–50 years age group, the period in which productive activities must be performed at work, is considered to be included in the treatment boundary due to their self-awareness of the symptoms or by others. The increase in the diagnosis and pharmacotherapy of the adult population could play a pivotal role in lowering the socioeconomic cost of ADHD [24].

The male predominance decreased during the study period in both the diagnosed (1.83 in 2015; 1.45 in 2018) and treatment population (1.74 in 2015; 1.39 in 2018). This finding is consistent with the published results [22].

Although the current study was outstanding in its analysis of the trend of adult ADHD using nationwide population data, several limitations need to be acknowledged. First, the ADHD diagnoses used in the study were derived from administrative claims data based on the International Classification of Disease 10th edition codes by physicians, rather than from structured clinical interviews. Second, the identified incidence rate in this study may be an underestimation of the actual incidence, as the study included only those who visited a clinic or hospital, that is, they had healthcare-seeking behavior. Third, caution is warranted when generalizing the results to other countries where no mandatory NHI system has been adopted.

## 5. Conclusions

Despite the above-mentioned limitations, the current study is among the few to date that have investigated the trends in the diagnostic and pharmacotherapy incidence of ADHD in the adult population.

## Figures and Tables

**Table 1:** The available medication for attention-deficit/hyperactivity disorder in Korea.

Methylphenidate	IR-MPH	Penid *
	ER-MPH	Medikinet *
		Metadate
	Long-MPH	Bisphentin
	OROS	Concerta *
Norepinephrine-reuptake inhibitor	Norepinephrine-reuptake inhibitor	Atomoxetine *
Norepinephrine–dopamine reuptake inhibitor	Norepinephrine-reuptake inhibitor	Bupropione *
α2-Adrenergic agonists	Norepinephrine-reuptake inhibitor	Clonidine

MPH—methylphenidate; IR—immediate release; ER—extended-release; OROS—osmotic-controlled release; * Approved for Adult ADHD.

**Table 2:** The diagnostic incidence of attention-deficit/hyperactivity disorder in adults.

		2015		2016		2017		2018		Test for Linear Trend	
Newly diagnosed		7762		9370		11,904		17,264		**0.0363**	positive
Total population (18 years old and above)		42,261,436		42,660,364		43,066,602		43,558,643			
Diagnostic Incidence		0.02%		0.02%		0.03%		0.04%			
		*n*	%	*n*	%	*n*	%	*n*	%	*p*	
Sex											
	Male	5020	64.7%	6099	65.1%	7356	61.8%	10,230	59.3%	**0.0304**	negative
	Female	2742	35.3%	3271	34.9%	4548	38.2%	7034	40.7%	**0.0451**	positive
Age											
	18–23	3717	47.9%	4392	46.9%	5073	42.6%	6843	39.6%	**0.0336**	negative
	24–30	1392	17.9%	1800	19.2%	2864	24.1%	4659	27.0%	**0.0384**	positive
	31–40	1028	13.2%	1260	13.4%	1738	14.6%	2890	16.7%	0.0551	positive
	41–50	652	8.4%	822	8.8%	987	8.3%	1486	8.6%	**0.0432**	positive
	51–60	332	4.3%	397	4.2%	494	4.1%	664	3.8%	**0.0230**	negative
	61 and over	641	8.3%	699	7.5%	748	6.3%	722	4.2%	0.1738	negative
	Mean (SD)	31.02	16.23	30.92	15.93	30.71	14.99	30.06	13.16		
Region											
	Others	3796	48.9%	4450	47.5%	5627	47.3%	8056	46.7%	**0.0393**	negative
	Urban	3966	51.1%	4920	52.5%	6277	52.7%	9208	53.3%	**0.0340**	positive
Insurance											
	NHI	7360	94.8%	8847	94.4%	11221	94.3%	16,474	95.4%	**0.0399**	positive
	Medical aid	402	5.2%	523	5.6%	683	5.7%	790	4.6%	**0.0027**	negative
Clinician’s specialty											
	Psychiatry	6428	82.8%	7763	82.8%	10,301	86.5%	15,956	92.4%	**0.0466**	positive
	Others	1334	17.2%	1607	17.2%	1603	13.5%	1308	7.6%	0.9356	negative
Hospital level											
	Hospital	2941	37.9%	3419	36.5%	3568	30.0%	4618	26.7%	0.0540	negative
	Private Clinic	4821	62.1%	5951	63.5%	8336	70.0%	12,646	73.3%	**0.0361**	positive

NHI: National Health Insurance. Bold: statistically significant value, *p* < 0.05.

**Table 3:** The treatment incidence of attention-deficit/hyperactivity disorder in adults.

		2015		2016		2017		2018		Test for Linear Trend	
Newly treated		3886		6040		8095		12,502		**0.0196**	positive
Total population (18 years old and above)		42,261,436		42,660,364		43,066,602		43,558,643			
Diagnostic Incidence		0.01%		0.01%		0.02%		0.03%			
		*n*	%	*n*	%	*n*	%	*n*	%	*p*	
Sex											
	Male	2471	63.6%	3841	63.6%	4929	60.9%	7273	58.2%	**0.0145**	negative
	Female	1415	36.4%	2199	36.4%	3166	39.1%	5229	41.8%	**0.0280**	positive
Age											
	18–23	1967	50.6%	2613	43.3%	3331	41.1%	4717	37.7%	**0.0187**	negative
	24–30	631	16.2%	1280	21.2%	2097	25.9%	3635	29.1%	**0.0213**	positive
	31–40	507	13.0%	913	15.1%	1277	15.8%	2214	17.7%	**0.0285**	positive
	41–50	298	7.7%	589	9.8%	690	8.5%	1083	8.7%	**0.0228**	positive
	51–60	171	4.4%	237	3.9%	303	3.7%	444	3.6%	**0.0209**	negative
	61 and over	312	8.0%	408	6.8%	397	4.9%	409	3.3%	0.2251	negative
	Mean (SD)	30.47	16.08	30.98	15.25	30.17	13.89	29.74	12.24		
Region											
	Others	1888	48.6%	2846	47.1%	3784	46.7%	5848	46.8%	**0.0219**	negative
	Urban	1998	51.4%	3194	52.9%	4311	53.3%	6654	53.2%	**0.0177**	positive
Insurance											
	NHI	3,675	94.6%	5747.00	95.1%	7,665	94.7%	11966.00	95.7%	**0.0211**	positive
	Medical aid	211	5.4%	293	4.9%	430	5.3%	536	4.3%	**0.0041**	negative
Clinician’s specialty											
	Psychiatry	3272	84.2%	5077	84.1%	7053	87.1%	11679	93.4%	**0.0297**	positive
	Others	614	15.8%	963	15.9%	1042	12.9%	823	6.6%	0.5142	negative
Hospital level											
	Hospital	1386	35.7%	1976	32.7%	2163	26.7%	3006	24.0%	**0.0271**	negative
	Clinic	2500	64.3%	4064	67.3%	5932	73.3%	9496	76.0%	**0.0204**	positive
Types of Medication at initiation	Penid	1308	33.7%	1358	22.5%	1361	16.8%	1644	13.1%	0.1457	negative
	Medikinet	18	0.5%	60	1.0%	68	0.8%	108	0.9%	**0.0272**	positive
	Bisphentin	0	0.0%	0	0.0%	3	0.0%	8	0.1%	0.0766	positive
	Concerta	680	17.5%	1593	26.4%	2291	28.3%	4081	32.6%	**0.0226**	positive
	Atomoxetine	336	8.6%	668	11.1%	788	9.7%	1055	8.4%	**0.0142**	negative
	Bupropione	271	7.0%	286	4.7%	317	3.9%	431	3.4%	0.0890	negative
	Clonidine	0	0.0%	51	0.8%	43	0.5%	46	0.4%	0.2878	positive
	Augmentation or combination	1273	32.8%	2024	33.5%	3224	39.8%	5129	41.0%	**0.0200**	positive

NHI: National Health Insurance. Bold: statistically significant value, *p* < 0.05.

## Data Availability

The data that support the findings of this study are available from the corresponding author upon reasonable request.
